# Combined antifungal effects of the vapor phases of *Zataria multiflora* and *Cinnamomum zeylanicum* essential oils against *Aspergillus flavus* and *Penicillium citrinum* in vitro and cheese

**DOI:** 10.1002/fsn3.3537

**Published:** 2023-06-28

**Authors:** Loqman Imaz, Javad Aliakbarlu, Lin Lin

**Affiliations:** ^1^ Faculty of Veterinary Medicine Urmia University Urmia Iran; ^2^ Department of Food Hygiene and Quality Control, Faculty of Veterinary Medicine Urmia University Urmia Iran; ^3^ School of Food and Biological Engineering Jiangsu University Zhenjiang China

**Keywords:** *C. zeylanicum*, cheese, essential oil, vapor phase, *Z. multiflora*

## Abstract

The objective of this study was to evaluate the antifungal effects of *Zataria multiflora* (ZEO) and *Cinnamomum zeylanicum* (CEO) essential oils in the vapor phase against *Aspergillus flavus* and *Penicillium citrinum* in vitro and cheese. The antifungal activities of the vapors of ZEO and CEO were assessed by determining the minimum inhibitory concentration (MIC), fractional inhibitory concentration (FIC) index, and inhibition zone diameters. Thymol (51.10%) and cinnamaldehyde (77.82%) were the main constituents of ZEO and CEO, respectively. The MIC values of the vapors of ZEO and CEO against *A. flavus* were 25 and 12.5 μL/L and against *P. citrinum* were 800 and 400 μL/L, respectively. The in vitro results showed that the combination of the vapor phases of ZEO and CEO could synergistically inhibit the growth of *A. flavus* (FIC index = 0.75). In the cheese, the growth of *P. citrinum* was entirely inhibited by the combination of ZEO and CEO vapors, even at very low concentrations (1/16 MIC). In conclusion, the vapor phases of ZEO and CEO showed the potential to be applied as effective natural antifungals and alternatives to synthetic preservatives in cheese.

## Graphical abstract

In this study, the antifungal activity of *Z. multiflora* (ZEO) and *C. zeylanicum* (CEO) essential oils vapors were examined. The vapor phases of ZEO and CEO showed significant antifungal effects. The combination of ZEO and CEO showed synergistic effect against *A. flavus* in vitro. The combination of ZEO and CEO could also synergistically inhibit *P. citrinum* in cheese.

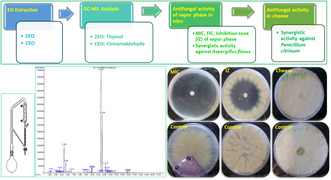

## 1 INTRODUCTION

Molds play an essential role in the spoilage of foods and may produce mycotoxins which threaten human health (Garnier et al., 2017; Kure & Skaar, 2019). *Penicillium* and *Aspergillus* are major genera of molds frequently found in cheese (Hlebová et al., 2022; López‐Díaz et al., 1996). Chemical preservatives are commonly used to control fungal and bacterial growth in cheese (European Parliament and Council, 2011). However, due to the potential side effects of chemical preservatives on human health and consumers' tendency to use chemical‐free foods, researchers have focused on finding effective and natural alternatives such as essential oils (EOs) (Carocho et al., 2015; Hlebová et al., 2022). EOs are volatile liquids with characteristic odors, and known biological activity extracted from different parts of aromatic plants (Burt, 2004). EOs or their components could protect the cheese from microbial damage and extend its shelf life (Valdivieso‐Ugarte et al., 2019). For example, it was reported that the addition of black cumin oil to the cheese decreased the inoculated pathogens (*Staphylococcus aureus*, *Escherichia coli*, *Listeria monocytogenes*, and *Salmonella* Enteritidis) up to 1.5 log CFU/g after 21 days of cold storage. Black cumin oil could also maintain the physicochemical and sensorial characteristics of the cheese (Hassanien et al., 2014).

*Zataria multiflora* Boiss. is an aromatic plant endemic to Iran, Pakistan, and Afghanistan; and locally known as “Avishane Shirazi” (Aliakbarlu et al., 2013; Shaiq Ali et al., 2000). *Z. multiflora* essential oil (ZEO) has various biological activities, including antioxidant, antibacterial, and antifungal activities (Aliakbarlu et al., 2013; Gandomi et al., 2009; Noori et al., 2012). Meanwhile, the antifungal activity of the liquid phase of ZEO against *Aspergillus flavus* in culture media and cheese model has also been reported (Moosavi‐Nasab et al., 2018). Cinnamon (*Cinnamomum zeylanicum*) essential oil (CEO) is rich in cinnamaldehyde and possesses antibacterial and antifungal effects (Mahmoudzadeh et al., 2022; Mortazavi & Aliakbarlu, 2019; Xing et al., 2010). The antimicrobial efficacy of the liquid phase of cinnamon EO in cheese has been shown (Jeong et al., 2014).

The strong and distinct odor of EOs in the liquid phase may have adverse effects on the sensory properties of foods, and then limit their application in foods (Khorshidian et al., 2018; Nazer et al., 2005). Meanwhile, the hydrophobic nature of EOs decreases their water solubility, and then limits their efficacy in food (Chen et al., 2014). One approach to overcome the drawbacks of the direct addition of the liquid phase of EOs to food is to use EOs in the vapor phase. The vapor phase of EOs may have a slight sensory impact (Khorshidian et al., 2018; Tyagi & Malik, 2011), and could be more effective than the liquid phase of EOs (Tyagi & Malik, 2011; Velázquez‐Nuñez et al., 2013). Meanwhile, the combination of EOs vapors with synergistic activity may reduce the amounts of EOs that are needed to show effective antimicrobial activity (Oh et al., 2022).

Antibacterial and antifungal effects of the liquid phase of many EOs have been reported (Bakkali et al., 2008; Burt, 2004; Lang & Buchbauer, 2012). However, less attention has been paid to the antimicrobial activity of the vapor phase of EOs. Due to the surface growth of fungi, using of EOs vapors may be considered as an exciting and effective alternative method to control fungal growth. Recently, the antifungal activity of the vapor phase of some EOs against *Penicillium commune* in cheese has been reported (Hlebová et al., 2022). However, to the best of our knowledge, no study so far evaluated the combined antifungal effects of the vapor phases of ZEO and CEO. Then, the present study aimed to examine the antifungal efficiency of ZEO and CEO vapor phases, alone and in combination, against *Aspergillus flavus* and *Penicillium citrinum* in culture media and cheese.

## 2 MATERIALS AND METHODS

### 2.1 Plant materials

The plant materials (*Zataria multiflora* and *Cinnamomum zeylanicum*) were purchased from a local grocery in Urmia, Iran, and their scientific names were confirmed by a botanist.

### 2.2 Essential oil extraction

The EOs were extracted from the leaves and stems of *Z. multiflora*, and from barks of cinnamon by the hydro‐distillation method using a Clevenger‐type apparatus. First, the ground plant materials (100 g) were added to a balloon containing 1000 mL of distilled water, and then heated for 3 h. The EOs were collected and dried by sodium sulfate and then stored in amber vials at 4°C until analysis. The extraction yields of ZEO and CEO were 1.5% and 2% (v/w), respectively.

### 2.3 Chemical analyses of EOs

The chemical constituents of EOs were determined using gas chromatography–mass spectrometry (GC–MS). The gas chromatograph (Agilent 7890A, USA) was equipped with the mass detector and a capillary column HP‐5 (30 × 0.25 mm ID, and 0.25 μm layer thickness). The chromatographic conditions were chosen according to a previously described method (Mortazavi & Aliakbarlu, 2019).

### 2.4 Preparation of fungal spore suspension

*Aspergillus flavus* (Persian Type Collection Culture (PTCC) 5006) and *Penicillium citrinum* (PTCC 5304) were obtained from the microbial collection of the Iranian Research Organization for Science and Technology. The fungi were cultured on a Saboroud dextrose agar (SDA) slant and incubated at 26 ± 1°C for 7–10 days. After that, 10 mL of distilled water was added, and then the surface of the agar was gently scraped by a sterile loop. The spore count of the suspension was measured using a Neobar slide. Finally, the spore count was adjusted to 10^6^ spore/mL with distilled water (Nguefack et al., 2004).

### 2.5 Determination of MIC

The minimum inhibitory concentration (MIC) of the vapor phases of ZEO and CEO against *A. flavus* and *P. citrinum* was determined using the disk volatilization method. First, 10 mL of molten SDA was poured on a plate (8 cm). After solidification of the agar, 10 μL fungal spore suspension (10^6^ spore/mL) was loaded in the center of the plate. To establish the paper disk on the plate lid, 1 mL of the molten agar was also poured on the center of the plate lid, and immediately, the paper disk was fixed on the agar by sterile forceps. ZEO and CEO were dissolved in ethyl acetate, and their different doses (0, 0.25, 0.5, 0.75, and 1 μL/disk against *A. flavus* and 0, 2, 4, 8, 16, 32, and 64 μL/disk against *P. citrinum*) were applied on the disks, respectively. These doses were nearly equal to the concentrations ranging from 0 to 25 μL/L against *A. flavus* and from 0 to 1600 μL/L against *P. citrinum*. After closing the lid of the plate, the plates were sealed using Parafilm. The plates were incubated at 26 ± 1°C for 5 days, and the colony growth was monitored daily. The MIC was expressed as a microliter of ZEO or CEO per volume (L) of air above the inoculated agar surface and defined as the lowest concentration of EO that inhibits colony formation after 5 days of incubation (Kloucek et al., 2012).

### 2.6 Determination of FIC index

The combined inhibitory effects of the vapor phases of ZEO and CEO at sub‐MIC concentrations (1/2 and 1/4 × MIC) were also measured as described above. The fractional inhibitory concentrations (FICs) were calculated according to the following formula:

$${\mathrm{FIC}}_{A}=\mathrm{MIC}\;\text{of}\;A\;\text{in the presence of}\;B/\mathrm{MIC}\;\text{of}\;A\;\text{alone}$$

$${\mathrm{FIC}}_{B}=\mathrm{MIC}\;\text{of}\;B\;\text{in the presence of}\;A/\mathrm{MIC}\;\text{of}\;B\;\text{alone}$$

$${\mathrm{FIC}}_{\text{Index}}={\mathrm{FIC}}_{A}+{\mathrm{FIC}}_{B}$$

The antifungal interactions were interpreted as synergistic (FIC_Index_ < 1), additive (FIC_Index_ = 1), and antagonistic (FIC_Index_ > 1) (Aguilar‐González et al., 2015).

The inhibition percentage of fungal growth on day 5 was also calculated according to the following equation:

$$\text{Inhibition of growth}\;\left(\%\right)=\left(C-T\right)/C\times 100$$

C and T indicate the colony diameter of the control and the treatment, respectively.

### 2.7 Determination of inhibition zone

In this test, 100 μL of fungal sore suspension (10^6^ spore/disk) was spread on SDA. Different doses of ZEO and CEO were loaded on the paper disks fixed on the center of the plate lid, respectively. Ethyl acetate was used as the negative control. The plates were sealed using parafilm and incubated at 26 ± 1°C for 5 days, and the diameters of the inhibition zone were measured each day and recorded as mm (Tyagi & Malik, 2011).

### 2.8 Antifungal activity in the cheese

Fresh Iranian white cheese (Pegah Dairy Co.; 60% moisture, 12.5% protein, 36% fat in dry matter, pH 4.5, *aw* = 0.96) was purchased from a local market in Urmia, Iran. The cheese was cut into slices with a thickness of 5 mm (equal to the thickness of agar on the plate), and placed into the plate. Then, 10 μL fungal spore suspension (10^6^ spore/mL) was loaded on the cheese surface in the center of the plate. After that, ZEO and CEO alone at MIC doses and in combination at sub‐MIC doses were poured on paper disks fixed on the lid of the plate. Finally, the plates were sealed with Parafilm and incubated at 26 ± 1°C for 5 days (Gandomi et al., 2009). The diameter of fungal growth was measured as mm each day. The inhibition percentage of fungal growth on day 5 was calculated by the formula given in Section 2.6.

### 2.9 Statistical analysis

All the experiments were carried out for at least three times. Analysis of variance (ANOVA) procedure and Duncan multiple range test were performed to analyze data using SPSS (version 18; Inc.) at the significant level of 0.05.

## 3 RESULTS AND DISCUSSION

### 3.1 Chemical composition of ZEO and CEO

The chemical constituents of ZEO and CEO are given in Tables 1 and 2, respectively. The major components of ZEO were thymol (51.10%), carvacrol (18.98%), p‐cymene (9.55%), and gamma‐terpinene (3.72%). Similar to this finding, several previous studies also reported that carvacrol and thymol were the main active substances of ZEO (Bazargani‐Gilani et al., 2014; Noori et al., 2012; Shafiee & Javidnia, 1997). Furthermore, it was shown that carvacrol and thymol could inhibit fungal growth (Pérez‐Alfonso et al., 2012).

**TABLE 1:** Chemical composition of *Zataria multiflora* essential oil (ZEO).

Compound	Area (%)
Alpha Thujene	0.14
Alpha Pinene	1.36
Beta Myrcene	0.66
Alpha Phellandrene	0.15
Alpha Terpinene	1.61
D‐Limonene	0.24
Beta Phellandrene	0.11
P‐Cymene	9.55
Gamma Terpinene	3.72
Sabinene Hydrate	0.12
Linalool	0.90
Terpinen‐4‐Ol	0.81
Alpha Terpinol	0.66
Thymol Methyl Ether	1.68
O‐Cymen‐5‐Ol	0.22
Thymol	51.10
Carvacrol	18.98
Caryophyllene	2.49
Cinnamaldehyde	1.35
Thymyl Acetate	0.41
Alpha Humulene	0.14
Ledene	0.27
Spathulenol	0.37
Caryophyllene Oxide	0.68

**TABLE 2:** Chemical composition of *Cinnamomum zeylanicum* essential oil (CEO).

Compound	Area (%)
Alpha Copaene	5.74
Cinnamaldehyde	77.82
Carvacrol	0.30
Gamma Muurolene	0.71
Beta Bisabolene	0.30
Alpha Curcumen	0.43
Alpha Muurolene	3.78
Alpha Amorphene	0.30
Υ‐Cadinene	0.31
Delta Cadinene	2.72
Cis Calamenene	1.52
Cinnamyl Acetate	0.16
Alpha Copaene	0.42
Ethyl Cinnamate	1.16

As seen from Table 2, cinnamaldehyde (77.82%), alpha‐copaene (5.74%), and alpha‐muurolene (3.78%) were determined as the main constituents of CEO. This result is in accordance with the findings reported in the previous studies (Mahmoudzadeh et al., 2022; Mortazavi & Aliakbarlu, 2019). The antimicrobial effect of cinnamon essential oil could be linked to cinnamaldehyde (Wang et al., 2005).

### 3.2 MIC of ZEO and CEO vapors

The MIC values of the vapor phase of ZEO and CEO against *A. flavus* and *P. citrinum* are summarized in Table 3. ZEO at a concentration of 1 μL/disk (25 μL/L) completely inhibited the fungal growth until day 5 of incubation. Then, the MIC of ZEO against *A. flavus* was determined as 25 μL/L. The doses lower than that also inhibited the fungal growth in the early days of incubation, while on day 5, 70%–86% inhibition was recorded compared to the control (data not shown). CEO at a concentration of 0.5 μL/disk (12.5 μL/L) totally inhibited the growth of *A. flavus*, and then this value was recorded as the MIC of CEO. As shown in Table 3, the MIC values of ZEO and CEO vapors against *P. citrinum* were 32 μL/disk (800 μL/L) and 16 μL/disk (400 μL/L), respectively.

**TABLE 3:** Minimum inhibitory concentration (MIC) of vapor phase of *Zataria multiflora* (ZEO) and *Cinnamomum zeylanicum* (CEO) essential oils against *A. flavus* and *P. citrinum*.

	MIC (μL/L)	
	*A. flavus*	*P. citrinum*
ZEO	25	800
CEO	12.5	400

The results concluded that the antifungal effect of CEO was stronger than ZEO against both fungi, which may be due to higher volatility and more potent antimicrobial activity of CEO components. In support of this, it was shown that the vapors of aldehyde components such as cinnamaldehyde were more active than terpenes such as thymol and carvacrol (Fancello et al., 2020). Our results also found that *A. flavus* was more susceptible than *P. citrinum* to the antifungal effect of both ZEO and CEO vapors.

The MIC of CEO against *A. flavus* was 12.5 μL/L. In accordance with our results, other researchers reported that the MIC value of cinnamon EO vapor against *A. flavus* and *P. islandicum* was 13.1 and 8.7 μL/L, respectively (López et al., 2007). This work also showed that the antifungal effect of the vapor of cinnamon oil was higher than oregano and thyme oils (López et al., 2007). It was reported that the MIC of the vapor phase of *Cinnamomum zeylanicum* EO against *P. corylophilum* was 0.1563 μL/L (Ji et al., 2019). The vapors of clove (92 μL/L) and mustard (15 μL/L) EOs could inhibit the growth of *Botrytis cinerea* (Aguilar‐González et al., 2015). Another study reported that 41.1 μL/L of *Brassica nigra* EO was needed to inhibit the growth of *P. citrinum*, *A. ochraceus*, and *A. niger* (Mejía‐Garibay et al., 2015). Other researchers evaluated the antifungal activity of the vapor phase of some selected EOs against *Penicillium commune*, and the results showed that litsea and clove EOs had the best inhibitory effect with the MIC values of 15.62 and 31.25 μL/L, respectively (Hlebová et al., 2022).

### 3.3 Combined antifungal effects of ZEO and CEO vapors

The combined inhibitory effects of ZEO and CEO vapors at sub‐MIC concentrations on *A. flavus* and *P. citrinum* growth and colony formation are presented in Table 4. The combination of ¼ MIC of ZEO with ½ MIC of CEO as well as ½ MIC of ZEO with ¼ MIC of CEO synergistically inhibited the growth of *A. flavus* (FIC_Index_ = 0.75). Meanwhile, ¼ MIC of ZEO in combination with ¼ MIC of CEO inhibited the growth of *A. flavus* until day 3 of incubation. In the case of *P. citrinum*, the combinations of ZEO and CEO vapors at sub‐MIC concentrations could not inhibit the fungal growth completely. However, the inhibition percentages from 43% to 66% were observed on day 5 of incubation.

**TABLE 4:** Combined inhibitory effects vapor phases of *Zataria multiflora* essential oil (ZEO) and *Cinnamomum zeylanicum* essential oil (CEO) on *A. flavus* and *P. citrinum* growth and colony diameter (mm).

	Incubation time (day)					I (%)
	1	2	3	4	5	
*A. flavus*						
Treatments						
1/2 MIC ZEO + 1/2 MIC CEO	–	–	–	–	–	100
1/4 MIC ZEO + 1/2 MIC CEO	–	–	–	–	–	100
1/2 MIC ZEO + 1/4 MIC CEO	–	–	–	–	–	100
1/4 MIC ZEO + 1/4 MIC CEO	–	–	–	8.0 ± 0.00	12.3 ± 0.58	84.6
Control	14.6 ± 0.57	32.7 ± 0.58	52.6 ± 0.57	70.6 ± 0.58	80.0 ± 0.00	0
*P. citrinum*						
Treatments						
1/2 MIC ZEO + 1/2 MIC CEO	–	2.3 ± 0.58^c^	3.0 ± 0.0^d^	4.3 ± 0.58^d^	4.3 ± 0.58^c^	66
1/4 MIC ZEO + 1/2 MIC CEO	2.7 ± 0.58^b^	3.0 ± 1.0^c^	4.3 ± 0.58^c^	5.0 ± 0.0^c,d^	5.0 ± 0.00^c^	61
1/2 MIC ZEO + 1/4 MIC CEO	3.0 ± 0.0^b^	4.7 ± 0.58^b^	5.0 ± 0.0^c^	6.0 ± 1.0^b,c^	7.0 ± 0.00^b^	46
1/4 MIC ZEO + 1/4 MIC CEO	3.0 ± 0.0^b^	5.3 ± 0.57^b^	6.3 ± 0.58^b^	7.0 ± 1.0^b^	7.3 ± 1.15^b^	43
Control	6.0 ± 1.0^a^	9.0 ± 1.0^a^	11.7 ± 0.58^a^	12.7 ± 0.58^a^	13.0 ± 0.0^a^	0

*Note*: –, no colony formation (no growth). I (%), inhibition percentage on day 5. The different small letters in each column indicate significant differences among treatments (*p* < .05).

The synergism between cinnamaldehyde and thymol or carvacrol has been shown previously (Zhou et al., 2007). Then, the synergism between CEO and ZEO in this study might be attributed to synergistic interaction among their major components. It was shown that there was a synergistic effect between cinnamon bark and citronella essential oil vapors against *P. corylophilum* in vitro (Ji et al., 2019). The synergistic antifungal effect between clove and mustard EO vapors has also been reported (Aguilar‐González et al., 2015). Another study showed that the combination of the vapor phase of cinnamon and clove EOs could synergistically inhibit some pathogenic bacteria (Goñi et al., 2009).

### 3.4 Inhibition zone diameter

The inhibition zone diameter of ZEO and CEO vapor phases and their combination against *A. flavus* are given in Table 5. All individual and combined treatments were able to produce the inhibition zone against *A. flavus*. The inhibition zones of ZEO at ½ MIC combined with ½ MIC of CEO were comparable with the inhibition zones of their MIC concentrations on day 5. However, the inhibition zones of other combinations were smaller than that of MIC alone.

**TABLE 5:** Inhibition zones diameter (mm) of *Zataria multiflora* essential oil (ZEO) and *Cinnamomum zeylanicum* essential oil (CEO) vapor phases and their combination against *A. flavus*.

Treatment	Incubation time (day)				
	1	2	3	4	5
MIC ZEO	36.7 ± 0.58^b,c^	26.7 ± 2.31^b^	24.3 ± 1.15^b^	22.3 ± 1.15^a^	21.3 ± 0.6^a^
MIC CEO	46.7 ± 0.57^a^	37.0 ± 0.0^a^	30.7 ± 0.58^a^	23.0 ± 0.0^a^	21.0 ± 1.73^a^
1/2 MIC ZEO + 1/2 MIC CEO	37.3 ± 0.57^b^	27.0 ± 0.0^b^	23.0 ± 0.0^b^	22.7 ± 0.6^a^	22.0 ± 0.0^a^
1/4 MIC ZEO + 1/2 MIC CEO	35.7 ± 0.58^c^	23.7 ± 1.15^c^	16.0 ± 0.0^c^	14.7 ± 0.6^b^	14.3 ± 0.6^b^
1/2 MIC ZEO + 1/4 MIC CEO	30.0 ± 0.0^d^	19.3 ± 0.58^d^	13.3 ± 0.57^d^	11.0 ± 0.0^c^	10.0 ± 0.0^c^
1/4 MIC ZEO + 1/4 MIC CEO	28.0 ± 1.0^e^	13.0 ± 1.0^e^	9.0 ± 1.73^e^	5.7 ± 0.58^d^	5.0 ± 0.0^d^
Control	+	+	+	+	+

*Note*: +, full growth (no inhibition zone). The different small letters in each column indicate significant differences among treatments (*p* < .05).

Table 6 shows the inhibition zone diameter of ZEO and CEO vapor phases and their combination against *P. citrinum*. CEO vapor at MIC concentration could produce the largest inhibition zone diameter (62 mm). Meanwhile, unlike ZEO, CEO at all sub‐MIC concentrations (1/2 MIC‐1/16 MIC) induced inhibition zone (data not shown). This finding suggests the stronger antifungal activity of CEO against *P. citrinum*. All individual and combined treatments could produce the inhibition zones against *P. citrinum*. However, the individual treatments were more effective than the combined ones against *P. citrinum*. This may be related to the weak antifungal effect of ZEO at sub‐MIC concentrations, which decreases the effectiveness of sub‐inhibitory combinations.

**TABLE 6:** Inhibition zones diameter (mm) of *Zataria multiflora* essential oil (ZEO) and *Cinnamomum zeylanicum* essential oil (CEO) vapor phases and their combination against *P. citrinum*.

Treatment	Incubation time (day)				
	1	2	3	4	5
MIC ZEO	44.7 ± 1.53^b^	44.0 ± 1.0^b^	43.0 ± 1.00^b^	41.6 ± 0.58^b^	38.0 ± 0.0^b^
MIC CEO	65.3 ± 2.52^a^	62.3 ± 0.58^a^	62.3 ± 0.57^a^	62.0 ± 0.0^a^	62.0 ± 1.0^a^
1/2 MIC ZEO + 1/2 MIC CEO	33.0 ± 1.0^c^	30.0 ± 1.0^c^	30.0 ± 1.0^c^	29.7 ± 0.57^c^	29.0 ± 1.0^c^
1/4 MIC ZEO + 1/2 MIC CEO	31.3 ± 0.58^c^	29.0 ± 1.0^c^	28.7 ± 0.58^d^	28.0 ± 0.0^d^	28.0 ± 0.0^c^
1/2 MIC ZEO + 1/4 MIC CEO	21.3 ± 1.52^d^	20.7 ± 0.58^d^	19.7 ± 0.58^e^	19.0 ± 0.0^e^	18.7 ± 0.58^d^
1/4 MIC ZEO + 1/4 MIC CEO	15.7 ± 1.15^e^	15.3 ± 0.57^e^	15.0 ± 0.0^f^	15.0 ± 0.0^f^	15.0 ± 0.0^e^
Control	+	+	+	+	+

*Note*: +, full growth (no inhibition zone). The different small letters in each column indicate significant differences among treatments (*p* < .05).

### 3.5 Antifungal effects of ZEO and CEO vapors in cheese

The inhibitory effects of ZEO and CEO vapors on the colony growth of *A. flavus* in the cheese during storage at 26 ± 1°C for 5 days are shown in Table 7. CEO vapor at MIC concentration was the most potent treatment in inhibiting *A. flavus* growth on the cheese surface. Interestingly, all combinations at sub‐MIC levels were more effective than MIC of ZEO in the prevention of *A. flavus* growth in cheese.

**TABLE 7:** Inhibitory effects of vapor phases of *Zataria multiflora* essential oil (ZEO) and *Cinnamomum zeylanicum* essential oil (CEO) on colony growth (mm) of *A. flavus* in cheese during storage at 26 ± 1°C.

Treatments	Storage day					I (%)
	1	2	3	4	5	
MIC ZEO	–	–	5.3 ± 0.58^b^	12.0 ± 1.0^b^	19.7 ± 1.53^b^	25.9
MIC CEO	–	–	–	–	5.0 ± 1.0^e^	81.5
1/2 MIC ZEO + 1/2 MIC CEO	–	–	–	4.3 ± 0.58^e^	12.0 ± 0.0^d^	55.5
1/4 MIC ZEO + 1/2 MIC CEO	–	–	–	6.7 ± 1.15^d^	13.7 ± 0.58^c,d^	49.6
1/2 MIC ZEO + 1/4 MIC CEO	–	–	5.3 ± 0.58^b^	9.0 ± 0.0^c^	15.3 ± 1.52^c^	38.5
1/4 MIC ZEO + 1/4 MIC CEO	–	–	4.3 ± 1.15^b^	10.0 ± 0.0^c^	20 ± 1.0^b^	27.1
Control	–	8.0 ± 1.0	13.3 ± 0.58^a^	19.7 ± 1.53^a^	27.0 ± 0.0^ab^	0

*Note*: –, no colony formation (no growth). I (%), inhibition percentage on day 5. The different small letters in each column indicate significant differences among treatments (*p* < .05).

Table 8 displays the antifungal effects of vapor phases of ZEO and CEO against *P. citrinum* in cheese. All individual and combined treatments could completely (100%) inhibit the growth of *P. citrinum* in cheese stored at 26 ± 1°C for 5 days. The combination of ZEO and CEO vapors at very low concentrations (1/16 MIC of ZEO + 1/16 MIC of CEO) was also able to complete the inhibition of *P. citrinum* growth in the cheese. Then, lower concentrations of ZEO and CEO vapors are needed to control the fungal growth in the cheese compared to the culture media. Based on the results, it can be concluded that the antifungal effects of the vapor phases of ZEO and CEO in the cheese were stronger than those in culture media. However, the mechanisms involved in this observation remain unclear. These findings are not in accordance with the results of other previous studies, which showed that higher levels of EOs in the liquid phase are required to inhibit microbial growth in foods compared to culture media (Gandomi et al., 2009; Noori et al., 2012), and it is due to interaction between food components and EOs constituents (Mahmoudzadeh et al., 2022; Ultee et al., 1998).

**TABLE 8:** Inhibitory effects of vapor phases of *Zataria multiflora* essential oil (ZEO) and *Cinnamomum zeylanicum* essential oil (CEO) on colony growth (mm) of *P. citrinum* in cheese during storage at 26 ± 1°C.

Treatments	Storage day					Inhibition (%)
	1	2	3	4	5	
MIC ZEO	–	–	–	–	–	100
MIC CEO	–	–	–	–	–	100
1/2 MIC ZEO + 1/2 MIC CEO	–	–	–	–	–	100
1/4 MIC ZEO + 1/2 MIC CEO	–	–	–	–	–	100
1/2 MIC ZEO + 1/4 MIC CEO	–	–	–	–	–	100
1/4 MIC ZEO + 1/4 MIC CEO	–	–	–	–	–	100
1/8 MIC ZEO + 1/4 MIC CEO	–	–	–	–	–	100
1/4 MIC ZEO + 1/8 MIC CEO	–	–	–	–	–	100
1/8 MIC ZEO + 1/8 MIC CEO	–	–	–	–	–	100
1/16 MIC ZEO + 1/8 MIC CEO	–	–	–	–	–	100
1/8 MIC ZEO + 1/16 MIC CEO	–	–	–	–	–	100
1/16 MIC ZEO + 1/16 MIC CEO	–	–	–	–	–	100
Control	–	–	12.3 ± 0.58	14.0 ± 1.15	16.0 ± 1.0	0

*Note*: –, no colony formation (no growth). I (%), inhibition percentage on day 5.

Many studies investigated the preservative effect of the liquid phase of EOs in different cheeses, and the promising results were achieved. However, due to the negative sensory impacts of EOs, their usage in cheese is still scarce (Christaki et al., 2021). One alternative method to overcome this problem is using EOs in the vapor phase, especially in combined form. In the present study, we evaluated the combined antifungal effects of the vapor phases of *Zataria multiflora* and *Cinnamomum zeylanicum* EOs against *A. flavus* and *P. citrinum*.

Unlike the liquid phase of EOs, a few works studied the antimicrobial effect of EOs vapor in cheese. In one of the limited number of studies, the antibacterial effect of the gaseous phase of EO of *Citrus limon* leaf against *Listeria monocytogenes* on *ricotta salata* cheese has been evaluated. The results showed that the combination of the gaseous phase of the EO and refrigeration temperature (5°C) significantly decreased *L. monocytogenes* in *ricotta salata* cheese (Fancello et al., 2020). Furthermore, a recent work evaluated the antifungal effect of the vapor phase of some EOs against *P. commune* on cheese, and the EOs of litsea and clove showed the most potent inhibitory activity (Hlebová et al., 2022).

However, other studies reported the antifungal effects of the liquid phase of EOs in cheese (Gandomi et al., 2009; Jeong et al., 2014; Noori et al., 2012). The antifungal effect of *Z. multiflora* EO in the liquid phase against *A. flavus* in culture media and Iranian white cheese has been investigated (Gandomi et al., 2009). The EO completely inhibited the fungal growth in culture media at concentrations higher than 400 ppm. In the cheese, however, the EO had partial inhibitory effects on the fungal growth at all concentrations tested (50–1000 ppm). Another similar study evaluated the antifungal activity of the liquid phase of *Z. multiflora* EO against *P. citrinum* in culture media and mozzarella cheese (Noori et al., 2012). The EO completely inhibited the fungal growth on potato dextrose agar at 200 ppm. However, the EO showed limited inhibitory effects against fungal growth on cheese at all concentrations examined (50–1000 ppm). Other authors also investigated the antifungal effect of the liquid phases of clove and *Z. multiflora* EOs on the growth of *A. flavus* in Iranian white cheese. The results showed that clove EO at a concentration of 150 ppm could completely inhibit *A. flavus* growth in the cheese, but *Z. multiflora* EO at a concentration of 600 ppm showed 91.3% inhibition (Moosavi‐Nasab et al., 2018). The in vitro antifungal activity of the liquid phases of different essential oils (cinnamon leaf or bark, basil, ginger, lemon, peppermint, pine needle, and spearmint) against *Penicillium* spp. has been evaluated (Jeong et al., 2014). The results showed that cinnamon leaf and bark EOs had the most potent antifungal activity. The results also indicated that cinnamon EO at a concentration of 10% (v/v) showed excellent antimicrobial activity in Appenzeller cheese.

## 4 CONCLUSIONS

In this study, we evaluated the antifungal performance of ZEO and CEO in the vapor phase against *A. flavus* and *P. citrinum* in the culture media and cheese. The in vitro results showed that the antifungal activity of the vapor phase of CEO against both fungi was more potent than that of ZEO. Meanwhile, *P. citrinum* was more resistant than *A. flavus* to the antifungal effect of both EOs. Results indicated that the combined use of ZEO and CEO vapors synergistically inhibited the growth of *A. flavus*. In the cheese, ZEO and CEO vapors, alone and in combination, showed inhibitory effects on *A. flavus* in the early days of storage, while their effects decreased at the end of storage. Interestingly, the combinations of ZEO and CEO vapors, even at very low levels (1/16 MIC), could synergistically inhibit the growth of *P. citrinum* on cheese. In conclusion, the vapor phases of ZEO and CEO could be potentially applied as natural and effective antifungals in cheese, with no impact on sensory quality.

## AUTHOR CONTRIBUTIONS

**Loqman Imaz:** Formal analysis (equal); investigation (equal); methodology (equal). **Javad Aliakbarlu:** Conceptualization (equal); data curation (equal); investigation (equal); software (equal); supervision (equal); validation (equal); visualization (equal); writing – original draft (equal). **Lin Lin:** Writing – review and editing (equal).

## CONFLICT OF INTEREST STATEMENT

The authors declare that there is no conflict of interest.

## Data Availability

The data that support the findings of this study are available from the corresponding author upon reasonable request.
